# Periodontitis Risk Variants at *SIGLEC5* Impair ERG and MAFB Binding

**DOI:** 10.1177/00220345211049984

**Published:** 2021-12-02

**Authors:** R. Mueller, A. Chopra, H. Dommisch, A.S. Schaefer

**Affiliations:** 1 Department of Periodontology, Oral Medicine and Oral Surgery, Institute for Dental and Craniofacial Sciences, Charité–University Medicine Berlin, corporate member of Freie Universität Berlin, Humboldt–Universität zu Berlin, and Berlin Institute of Health, Berlin, Germany; 2 Department of Biology, Chemistry and Pharmacy, Institute of Chemistry and Biochemistry, Freie Universität Berlin, Berlin, Germany

**Keywords:** oral inflammation, ERG transcription factor, MAFB transcription factor, mouth mucosa, enhancer elements, genetic

## Abstract

Periodontitis is a common complex inflammatory disease of the oral cavity. It is characterized by inflammation of gingival tissues and alveolar bone loss. Recently, a genome-wide association study and 2 genome-wide association study meta-analyses found 2 associated regions (haplotype blocks) at the inhibitory immune receptor gene *SIGLEC5* to increase the risk for periodontitis. The aims of the current study were the identification of the putative causal variants underlying these associations, characterization of their molecular biological effects, and validation of *SIGLEC5* as the target gene. We mapped the associated single-nucleotide polymorphisms to DNA elements with predictive features of regulatory functions and screened the associated alleles for transcription factor (TF) binding sites. Antibody electrophoretic mobility shift assays (EMSAs) with allele-specific probes were used to identify TF binding and to quantify allele-specific effects on binding affinities. Luciferase reporter assays were used to quantify the effect directions and allele-specific strength of the associated regulatory elements. We used CRISPR-dCas9 gene activation to validate *SIGLEC5* as a target of the association. EMSA in peripheral blood mononuclear cells showed that E-26 transformation–specific TF-related gene (ERG) binds at rs11084095, with almost complete loss of binding at the minor A-allele. Allele-specific reporter genes showed enhancer function of the DNA sequence at rs11084095, which was abrogated in the background of the A-allele. EMSA in B lymphocytes showed that TF MAF bZIP (MAFB) binds at the common G-allele of rs4284742, whereas the minor A-allele reduced TF binding by 69%, corresponding to 9-fold reduction of luciferase reporter gene activity by the A-allele. Using CRISPR-dCas9, we showed that the enhancer at rs4284742 strongly activated *SIGLEC5* expression, validating this gene as the target gene of the association. We conclude that rs11084095 and rs4284742 are putatively causal for the genome-wide significant associations with periodontitis at *SIGLEC5* that impair ERG and MAFB binding, respectively.

## Introduction

Periodontitis is a common complex inflammatory disease of the oral cavity and is characterized by chronic inflammation of the gingiva in conjunction with destruction of connective tissue and, subsequently, alveolar bone and tooth loss. Periodontitis has a range of manifestations that differ among individuals in severity and progression of tissue destruction and age of disease onset. The basis of phenotypic variation of complex diseases largely is the genetic variability among individuals (Timpson et al. 2018). The genetic variants mostly are single-nucleotide polymorphisms (SNPs; 1000 Genomes Project et al. 2015), and the number of functional SNPs and their biological consequences, magnitude of effects, and interactions with one another and with environmental factors shape disease manifestations (Boyle et al. 2017; Wray et al. 2018). To identify genetic susceptibility loci of periodontitis, various genome-wide association studies (GWASs) have been performed. Recently, a GWAS and 2 GWAS meta-analyses independently found SNPs at the inhibitory immune receptor gene sialic acid–binding Ig-like lectin 5 (*SIGLEC5*) to be associated at a genome-wide significance level (*P* < 5 × 10^-8^) with early-onset generalized stage III, grade C periodontitis (Munz et al. 2017) and more moderate late-onset forms (Munz et al. 2019; Shungin et al. 2019). Additionally, a study that integrated expression quantitative trait locus (eQTL) data with GWAS associations implied *SIGLEC5* as periodontitis risk gene (Li et al. 2020). All SNPs of these associations are located in the introns of *SIGLEC5.* These are regions that do not code for a protein, but variants in introns can change the activity of a gene or cause a protein to be produced in the wrong place or at the wrong time. The closest genes in a distance of the associated SNPs are *SIGLEC14* and *ZNF175.* eQTL effects for rs4284742 and rs11084095 suggested *SIGLEC5* as the target gene of the association, with the most significant eQTL effects detected in blood and monocytes, the tissue where *SIGLEC5* is almost exclusively expressed (except of placenta), with *P* = 7.7 × 10^–14^ and *P* = 6.4 × 10^–23^, respectively. Reported eQTLs of the associated SNPs were much weaker for other genes, implying *SIGLEC5* as the most likely target gene of the association. *SIGLEC5* is an inhibitory transmembrane receptor that binds sialic acids and sialic acid–containing glycan ligands, which are expressed on the surfaces of certain pathogens and bacteria. It is discussed that it mediates crosstalk of pathogen-associated molecular patterns (PAMPs) and danger-associated molecular patterns (DAMPs) during infection and wound healing (Pillai et al. 2012).

To elucidate the molecular mechanisms that predispose to increased disease susceptibility, genetic associations need to be leveraged to biological meaning. This poses a challenge because the most significant associated variant, called the GWAS lead SNP or sentinel variant, most often is not identical with the functional variants that caused the association. This is explained by the fact that numerous SNPs are in strong linkage disequilibrium (LD) and coinherited with the GWAS lead SNPs, comprising associated haplotype blocks (Gabriel et al. 2002).

The aims of the current study were to identify the putative causal variants of the associations with *SIGLEC5*, to characterize the role of the effect alleles in the disease etiology, and to validate *SIGLEC5* as the target gene of the association.

## Methods and Materials

Protocols for cell culture, transfection, quantitative reverse transcription polymerase chain reaction, isolation of peripheral blood mononuclear cells (PBMCs), and eQTL analysis are given in the appendix.

### Selection of Putative Causal Variants

To identify putative causal variants, we integrated data from ENCODE (ENCODE Project Consortium 2012) with the genomic locations of the associated SNP (Appendix). We determined all SNPs in strong LD (*r*^2^ > 0.8) to the GWAS lead SNPs (Munz et al. 2017; Shungin et al. 2019) in the northwestern European populations CEU (Utah residents with northern and western European ancestry) and GBR (British in England and Scotland; 1000 Genomes Project et al. 2010) of the International Genome Sample Resource using the online tool LDproxy (Machiela and Chanock 2015). Subsequently, sequence to motif alignments for these SNPs were performed with various libraries of binding matrixes for transcription factor binding sites (TFBSs) from Transfac professional (geneXplain), SNPInspector (Genomatix), and the open access database Jaspar. The transcription factor (TF) binding motif was confirmed via the web interface for position weight matrix (PWM) model generation and evaluation PWMTools (Ambrosini et al. 2018).

### Electrophoretic Mobility Shift Assay

To determine allele-specific protein-DNA binding, the Gelshift Chemiluminescent EMSA Kit (Activemotif) was used. The nuclear protein extract of Raji cells was extracted as described in the Appendix. For the supershift binding reaction, Raji cells or PBMC nuclear extract (10 µg) and biotin-labeled double-stranded oligonucleotides (20 fmol) were incubated at room temperature for 20 min with 1× binding buffer and 2 µL of specific antibody. To verify the result of DNA-protein interaction, unlabeled oligonucleotides (4 pmol) were added to the binding reaction. The reactions were loaded onto a 5% native polyacrylamide gel and run in 0.5× TBE buffer at 100 V for 1 to 1.5 h. After electric transfer of the products to a nylon membrane, the membrane was cross-linked at 120 mJ/cm^2^; the biotin-labeled oligonucleotides were detected by chemiluminescence; and the absolute value area of the shifted bands was quantified by ImageJ. All electrophoretic mobility shift assays (EMSAs) were performed in triplicates. Sequences of oligonucleotide probes, antibodies, and analysis details are given in the Appendix.

### Luciferase Reporter Gene Assay

Cloning and transfection of the luciferase reporter gene experiments are described in the appendix. All transfections were performed in 3 independent biological replicates.

### CRISPR-dCas9 Activation

CRISPR-dCas9 activation (CRISPRa) provides the possibility to test whether a genomic site serves as a cis-regulatory element for a target gene of interest (Simeonov et al. 2017). It allows specific and efficient quantification of the regulatory potential that a chromatin sequence has on gene expression in the endogenous context, including naturally occurring variants. We used CRISPRa to analyze if the DNA elements at rs4284742 and rs11084095 had regulatory effects on *SIGLEC5* expression. Details are given in the Appendix.

## Results

### Assignment of Putative Causal Associated Variants by Integration of LD and Regulatory Chromatin Features

**Figure 1.**
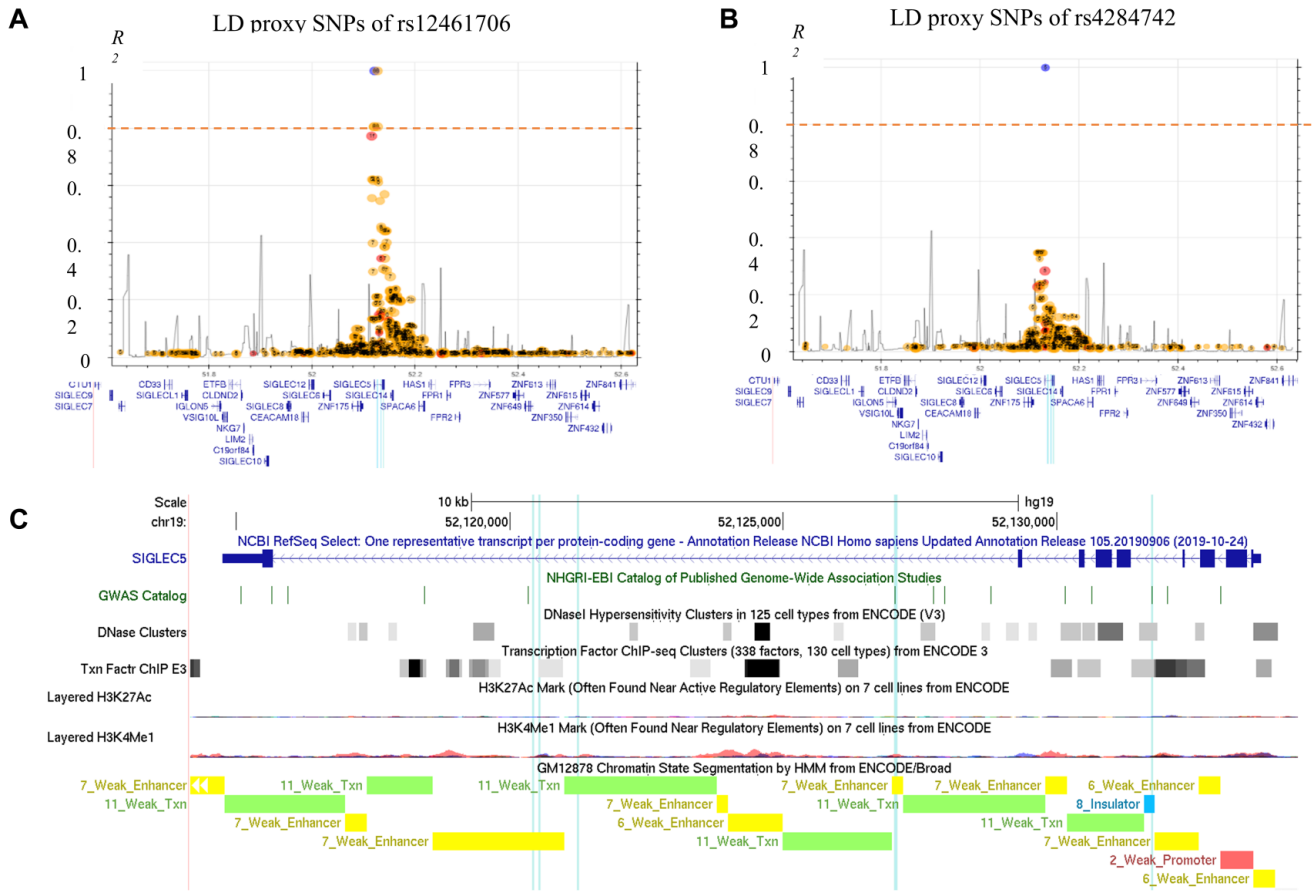
Linkage and chromosomal positions of the single-nucleotide polymorphisms (SNPs) that showed genome-wide significant associations with increased periodontitis susceptibility. (**A**) rs12461706 shows strong linkage disequilibrium (LD) with 3 SNPs (*r*^2^ > 0.8). The plot was generated with rs12461706 as the index SNP with genotype data from the 1000 Genomes Project for populations CEU (Utah residents with northern and western European ancestry) and GBR (British in England and Scotland) in a 500,000–base pair window (LDproxy Tool). The dashed horizontal line indicates *r*^2^ = 0.8. The SNPs were aligned to their chromosomal positions (*x*-axis). The blue vertical line shows the position of the LD SNPs at *r*^2^ > 0.8. rs3829655 missed the LD threshold with *r*^2^ = 0.77. This SNP is a benign exonic synonymous SNP and was not included in the transcription factor binding site analysis. (**B**) rs4284742 is not in strong LD with other SNPs (*r*^2^ > 0.8). (**C**) rs4284742 locates at chromatin elements that correlate with regulatory functions of gene expression (open chromatin as determined by DNAse I hypersensitivity and transcription factor binding sites experimentally confirmed by ChIP-Seq), as determined in various cell types (data from ENCODE). Chromatin segmentation in B-lymphocyte cells (GM12878) indicated enhancer elements at rs34984145, rs11880807, and rs11084095. The GWAS lead SNPs rs12461706, rs11084095, and rs4284742 and their LD SNPs (*r*^2^ > 0.8) are highlighted with blue vertical lines. SNP order from left to right: rs34984145, rs11880807, rs12461706, rs11084095, rs4801882, and rs4284742. Because rs11084095 and rs4801882 have a distance of 24 base pairs, they are indicated as 1 line. This figure is available in color online.

The haplotype block tagged by the GWAS lead SNPs were relatively narrow and encompassed few intronic cosegregating SNPs (Fig. 1A, B). The closest genes in a distance of the associated SNPs are *SIGLEC14* and *ZNF175* (Appendix Fig. 1). rs4284742 (Munz et al. 2017) had no other variant in strong LD (*r*^2^ > 0.8), whereas the adjacent associated haplotype block that was tagged by the GWAS meta-analyses’ lead SNPs rs12461706 (Shungin et al. 2019) and rs11084095 (Munz et al. 2019) comprised 5 SNPs in strong LD. These included the 2 sentinel variants and 3 additional tagging SNPs (Table). Of the 6 SNPs that tagged the 2 haplotype blocks, only rs4284742 mapped to regulatory DNA elements as determined by ChIP-Seq and DNAse I hypersensitivity. rs11084095, rs34984145, and rs11880807 located to a region that showed H3K4Me1 methylation in the B-cell line GM12878, a methylation mark that is enriched at active and primed cell type–specific enhancers. Both haplotype blocks were separated from one another by an insulator element (Fig. 1C).

**Table.** SNPs in Linkage Disequilibrium (*r*^2^ > 0.8) with the GWAS Lead SNPs rs4284742, rs12461706, and rs11084095.

	Location (GRCh37/hg19)	Allele			*r*^2^ with rs12461706		
SNP ID		Common	Minor	MAF^a^		GWAS Lead SNP	Predicted TFBS^b^
**rs4284742**	chr19:52131733	G	A	0.25	0.19	Munz et al. (2017)	MAFB (82%)
**rs12461706**	chr19:52121235	A	T	0.40	1.00	Shungin et al. (2019)	None
**rs11084095**	chr19:52127030	G	A	0.40	1.00	Munz et al. (2019)	ERG (93%)
rs11880807	chr19:52120522	A	G	0.30	0.81		None
rs34984145	chr19:52120410	A	T	0.43	0.81		BACH2 (83%)
rs4801882	chr19:52127053	G	A	0.44	0.81		None

Bold indicates GWAS-lead SNPs.

GWAS, genome-wide association study; MAF, minor allele frequency; SNP, single-nucleotide polymorphism; TFBS, transcription factor binding site.

a Indicated for CEU (Utah residents with northern and western European ancestry). MAFs for different human ethnicities were similar to northwest Europeans.

b Position weight matrix similarity score.

**Figure 2.**
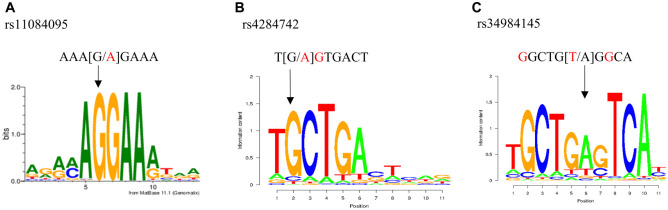
rs11084095 and rs4284742 have allele-specific effects on ERG and MAFB binding. (**A**) The ERG transcription factor (TF) binding motif has a matrix similarity of 93% with the DNA sequence at the common G-allele of rs11084095. (**B**) The MAFB TF binding motif shows a matrix similarity of 82% with the DNA sequence at the common G-allele of rs4284742. (**C**) For the common A-allele of rs34984145, a transcription factor binding site was predicted by SNPInspector for the TF BACH2 with a matrix similarity of 83%, whereas the rare T-allele strongly reduces binding affinity.

We investigated PWM libraries whether the nucleotide variants of the 6 SNPs changed predicted TFBSs. For the common allele of rs11084095, a TFBS for the ETS transcription factor ERG with a matrix similarity of 93% was predicted (Fig. 2A); for the common allele of rs4284742, a TFBS for the TF MAF bZIP transcription factor B (MAFB) with a matrix similarity of 82% was predicted (Fig. 2B); and for the common allele of rs34984145, a TFBS for BACH2 with a matrix similarity of 83% was predicted (Fig. 2C). PWMTools confirmed the high similarity to the TF binding motifs at these SNPs. At the noneffect alleles of the other SNPs, no TFBS was predicted. We selected the 3 SNPs rs4284742, rs11084095, and rs34984145 for subsequent validation of TF binding in vitro.

### TFs ERG and MAFB Show Allele-Specific Binding at rs11084095 and rs4284742

**Figure 3.**
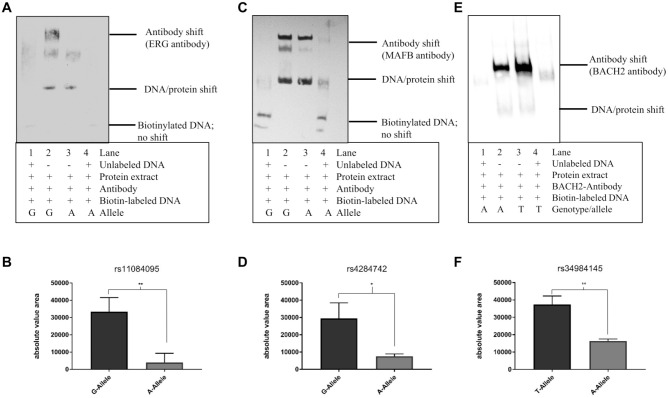
Transcription factor binding site of BACH2 at rs34984145. (**A**) ERG antibody electrophoretic mobility shift assay (EMSA) performed with rs11084095 allele-specific oligonucleotide probes and nuclear protein extract from peripheral blood mononuclear cells. (**B**) Absolute value area of the antibody-specific bands. In the background of the rare A-allele of rs11084095, ERG binding to the allele–specific oligonucleotides was 99% reduced compared to the common G-allele (*P* = 0.005). (**C**) MAFB antibody EMSA performed with rs4284742 allele-specific oligonucleotide probes and nuclear protein extract from Raji cells. (**D**) Absolute value area of the antibody-specific bands. In the background of the rare A-allele of rs4284742, MAFB binding to the allele-specific oligonucleotides was 69% reduced (*P* = 0.02) as compared with the common G-allele. (**E**) BACH2 antibody EMSA performed with rs34984145 allele-specific oligonucleotide probes and nuclear protein extract from Raji cells. (**F**) Absolute value area of the antibody-specific bands. In the background of the common A-allele of rs34984145, BACH2 binding to the allele-specific oligonucleotides was reduced by 59% (*P* = 0.007) as compared to the rare T-allele. Binding of ERG, MAFB, and BACH2 antibodies to allele-specific probes is shown in lanes 2 and 3. Probes with the effect allele abrogated antibody binding as compared with probes with the common G-allele. Unlabeled DNA was added to verify that the band shift was antibody specific in lanes 1 and 4. Values are presented as mean ± SD. *P < .05. **P < .01.

To prove protein binding at the 3 selected SNPs and to give evidence for allele-specific binding of the predicted TFs, we performed an EMSA using ERG, MAFB, and BACH2 antibodies with allele-specific DNA probes. *SIGLEC5* is mainly expressed in various lymphocytes of the innate immune system and in B cells (Crocker and Varki 2001). We performed the EMSA for rs11084095 with protein extract from PBMCs because in addition to *SIGLEC5*, ERG is weakly expressed in this cell type, too. MAFB is expressed in whole blood (including B lymphocytes), and BACH2 is expressed in B lymphocytes. Therefore, we performed the EMSA for rs4284742 and rs34984145 with protein extract from a B-lymphocyte cell line (Raji cells). In each EMSA we observed a band supershift with the TF-specific antibody and allele-specific oligonucleotides (Fig. 3A–F). The background of the rare A-allele of rs11084095 significantly reduced ERG binding with *P* = 0.005 to 99% as compared with binding at the common G-allele (Fig. 3A, B). The background of the rare A-allele of rs4284742 significantly reduced MAFB binding with *P* = 0.02 to 69% compared to binding at the common G-allele (Fig. 3C, D). At the common A-allele of rs34984145, BACH2 binding showed a significant reduction with *P* = 0.007 of TF binding by 59% as compared with the rare T-allele (Fig. 3E, F).

### rs4284742, rs11084095, and rs34984145 Are Located in Transcriptional Activators

**Figure 4.**
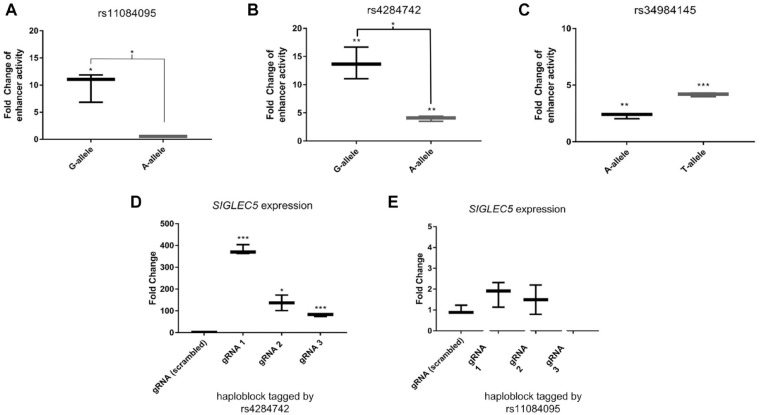
rs11084095 and rs4284742 have allele-specific effects on luciferase activity. The 65-bp DNA sequence up- and downstream of rs11084095 and the 75-bp DNA sequence spanning rs4284742 showed allele-specific enhancer activity in HeLa cells. (**A**) In the background of the common rs11084095 G-allele, luciferase activity was 9.9-fold increased (*P* = 0.015) versus the rare A-allele, which showed no upregulation. The difference between alleles was significant with *P* = 0.013. (**B**) The common rs4284742 G-allele increased luciferase activity 13-fold (*P* = 0.008) as compared with the rare A-allele, which increased the activity 4-fold (*P* = 0.002). The differences between alleles was significant with *P* = 0.010. (**C**) Both alleles of rs34984145 showed significant enhancer activity in HeLa cells. The rare T-allele showed stronger upregulation of 4.2-fold (*P* = 0.0001) versus the common A-allele, which showed an upregulation of 2.3-fold (*P* = 0.002). The difference between alleles was not significant. (**D**) The haplotype block at rs4284742 has a cis-regulatory effect on *SIGLEC5* expression. CRISPRa of the genomic region at rs4284742 induced *SIGLEC5* expression as compared with the scrambled gRNA. gRNA 1 (chr19:52131631-52131649), gRNA 2 (chr19:52131441-52131459), and gRNA 3 (chr19:52131754-52131773) induced *SIGLEC5* expression 380-fold (*P* = 0.00008), 137-fold (*P* = 0.03), and 80-fold (*P* = 0.0009), respectively (positions are given for genome build GRCh37/hg19). (**E**) CRISPRa of the genomic region at rs11084095 did not significantly induce *SIGLEC5* expression (gRNA 1: chr19:52126982-52127000, gRNA 2: chr19:52127105-52127123, gRNA 3: 2126912-52126930). Error bars indicate 95% CI. *P < .05. **P < .01. ***P < .001.

Regulatory DNA elements can either activate or repress transcription. To measure the activity of the regulatory elements at rs11084095, rs4284742, and rs34984145 and to discriminate their effect directions and allele-specific effect sizes, we employed luciferase reporter gene assays. ERG is expressed in HeLa cells at similar levels as in PBMCs. Because HeLa cells can be transfected more efficiently than PBMCs, which improves the detection of differences in reporter gene expression, we performed the reporter gene experiments in HeLa cells. Here, luciferase activity showed a significant increase in the background of the common G-allele of rs11084095 by 9.9-fold (*P* = 0.015) as compared with the empty plasmid. The rare A-allele showed no increase in luciferase activity. The difference between alleles was significant with *P* = 0.013 (Fig. 4A).

The DNA sequence with the common G-allele of rs4284742 showed 13-fold upregulation of luciferase activity (*P* = 0.008) in HeLa cells as compared with the reporter gene without this regulatory element (Fig. 4B). The reporter gene with the minor A-allele showed an upregulation of 4-fold (*P* = 0.002). The difference between alleles was significant with *P* = 0.01.

The luciferase gene activity of rs34984145 showed weak but significant upregulation for both alleles (T-allele: 4.2-fold, *P* = 0.0001; A-allele: 2.3-fold, *P* = 0.002), but the fold change difference between alleles was <2 (Fig. 4C), indicating allele-specific effects of low biological relevance.

These experiments indicated that the regulatory elements act as enhancers. Additionally, these experiments showed strong allele differences of rs11084095 and rs4284742 on enhancer activity.

### CRISPR-dCas9 Activation of rs4284742 Showed Cis-regulation of *SIGLEC5*

For rs11084095, an eQTL effect on the expression of *SIGLEC5* was reported in monocytes with *P* = 6.4 × 10^–23^ (Zeller et al. 2010), and for rs4284742, an eQTL effect on the expression of *SIGLEC5* was noted in whole blood with *P* = 7.7 × 10^–14^ (Blood eQTL browser; Westra et al. 2013), suggesting that the disease-associated elements have cis-regulatory effects on the expression of *SIGLEC5* (Appendix Tables 1 and 2). To validate the regulatory potential of the associated haplotype blocks tagged by rs11084095 and rs4284742, we performed CRISPRa in HeLa cells because Raji cells showed poor survival after transfection with the CRISPRa plasmids, probably because of DNA toxicity (Kim et al. 2014). CRISPRa of rs4284742 (gRNAs located in 20- to 300-bp distance from the SNP) with the synergistic activation mediator (SAM) system strongly increased the expression of *SIGLEC5* as compared with cotransfected unspecific control sgRNAs (FC = 380, Fig. 4D; Appendix Fig. 2). This proved that the DNA element at rs4284742 exerts a strong cis-regulatory effect on *SIGLEC5* and implied that *SIGLEC5* is the target gene of the disease-associated genetic variant. CRISPRa of rs11084095 with 3 gRNAs (25- to 120-bp distance from the SNP) showed no significant upregulation of *SIGLEC5* (Fig. 4E).

Because the alleles of rs34984145 showed no different effects on luciferase activity and no bloodborne eQTLs of rs34984145 on the expression of *SIGLEC5* were reported, we considered that rs34984145 is not a causal variant of the association and did not perform CRISPRa for this SNP.

## Discussion

Two large GWASs independently found genetic variants at *SIGLEC5* to be associated with different forms of periodontitis, indicating broad relevance of this locus for the disease etiology. We identified rs4284742 and rs11084095 as putative causal variants of the associations with early-onset generalized stage III, grade C periodontitis (Munz et al. 2017) and with more moderate forms (Shungin et al. 2019), respectively. Both SNPs were not in LD (*r*^2^ = 0.2) and located on different haplotype blocks. However, rs12461706 was in complete LD (*r*^2^ = 1) with rs11084095, the lead SNP of a GWAS-meta-analysis that combined the generalized stage III, grade C periodontitis cases with a GWAS sample of more moderate periodontitis (Teumer et al. 2013), which was also included in the GWAS meta-analysis of Shungin et al. (2019). To identify the putative causal variants of these associations, we analyzed all SNPs that were in strong LD to the GWAS lead SNPs for locating to predicted TFBSs. The 2 complementary TF databases of Transfac professional and SNPInsepctor allowed prediction of binding sites of all human TFs currently known. However, it is possible that we missed a TF with no currently known binding site. Another possible limitation was that we confined the analysis to SNPs that indicated strong linkage according to *r*^2^ > 0.8. We used this LD measure because the *r*^2^ coefficient of correlation takes account of allele frequency. Strong LD indicated by D′ but not by *r*^2^ would include alleles that are inherited with the particular GWAS lead SNP but are not carried by the majority of cases because they are rare. Such alleles would not be suggestive as causative variants because they would not explain the association for most cases. However, this does not exclude the existence of rare susceptibly variants at *SIGLEC5*, but such variants have no disease-relevant role for the general population. Instead, their effects would become noticeable in individual cases.

At the position of rs11084095, we identified a binding site for the TF ERG with 93% PWM matrix similarity. We showed that the sequence at rs11084095 is a binding site for ERG and has enhancer activity. The background of the rare effect allele significantly reduced ERG binding and enhancer activity. ERG is an essential TF for endothelial homeostasis, a system control state that encompasses acute responses to injury to support repair of damaged endothelium (Heiss et al. 2015). Changes in endothelial homeostasis (i.e., endothelial injury) affect the vascular structure by interacting with extracellular matrix turnover and influence endothelial membrane function and adhesiveness to proteins of the coagulation cascade and platelets, membrane permeability, and integrity (Gulino-Debrac 2013). Corresponding to function, ERG is mainly expressed in endothelial cells but also in leukocytes (Uhlen et al. 2015). The GWAS catalog (Welter et al. 2014) reports that genetic variations of ERG are associated with numerous blood cell traits and blood pressure, as well as bone mineral density and osteoarthritis. Notably, it was recently shown that soluble SIGLEC5 appears efficient in blocking leukocyte rolling over P-selectin (platelet selectin) and E-selectin (endothelial selectin; Pepin et al. 2016). In human endothelial cells, ERG associates with enhancers of von Willebrand factor (Kalna et al. 2019) and von Willebrand factor may act as a ligand for SIGLEC5 (Pegon et al. 2012), suggesting a possible link between endothelial homeostasis and SIGLEC5 function.

At the position of SNP rs4284742, we identified a predicted TFBS of the TF MAFB and showed that the effect allele, which is the common allele, provided strong MAFB binding affinity as compared with the noneffect allele. Additionally, we showed that CRISPR-dCas9 activation of the genomic sequences at rs4284742 activated *SIGLEC5* expression. These experiments indicated that MAFB regulation is linked with activation of *SIGLEC5* expression and with increased risk for early-onset periodontitis. MAFB belongs to the subfamily of the large Maf transcription factors (Santos-Gallego 2016). It is expressed by monocytes and is required for differentiation to macrophages (Kelly et al. 2000; Gemelli et al. 2006). Furthermore, it negatively regulates osteoclast generation via inhibition of the transcription factor NFATc1 and the osteoclast-associated receptor (OSCAR; Kim et al. 2007), implying a functional context of the genetic association with osteoclast differentiation. Additionally, MAFB was shown to promote sprouting angiogenesis (Jeong et al. 2017). During healing of tissue injuries, angiogenic capillary sprouts invade the wound clot and organize into a microvascular network throughout the granulation tissue (reviewed by Tonnesen et al. 2000).A challenge for healing of aseptic tissue injuries is discrimination from infections. This is achieved by the innate immune system through danger- or pathogens-associated molecular patterns, DAMPs and PAMPs. Sialoside-based pattern recognition by SIGLEC5 receptors was shown to selectively suppress the immune response to DAMPs, suggesting a mechanism by which aseptic tissue injury and infection are distinguished (Chen et al. 2009). This mechanism is normally well controlled to avoid autoimmune destruction. However, many viruses and pathogenic bacteria express sialidase as virulence factors (Crennell et al. 1993; reviewed by Drzeniek 1972; Crennell et al. 1993), and microbial-expressed sialidases might have potential to abrogate the SIGLEC-mediated inhibitory effects of DAMPs on the innate immune system during healing of injured tissues. As a result, DAMPs and PAMPs would become indistinguishable, which could provide an explanation for massive inflammation and tissue destruction (Liu et al. 2009), as typically seen in severe periodontitis phenotypes (stage III) with a rapid rate of progression (grade C). This may explain why, in specific situations, *SIGLEC5* expression may increase the risk for alveolar bone loss.

Notably, the maximal PWM score of MAFB was 82%. We interpret the incomplete similarity score in a way that the specificity of protein-DNA binding depends not solely on the DNA sequence but also on the 3-dimensional structure of DNA and TF protein macromolecules (Rohs et al. 2010). This result in variation of functional binding motifs and, accordingly, the predicative accuracy of a PWM should be interpreted with caution (Weirauch et al. 2013). However, the rare allele further reduced the matrix similarity to 60%, which corresponded with reduced MAFB binding in the EMSA and reduced reporter gene activity. Using CRISPRa, we could show that the enhancer at rs4284742 regulates *SIGLEC5* expression. However, we lacked experimental evidence for such effects at rs11084095. This does not signify that *SIGLEC5* is not the target gene of this association. Notably, the strongest eQTL of rs11084095 affected *SIGLEC5* expression (e.g., *P* = 6.4 × 10^–23^ in monocytes; Zeller et al. 2010) but no other genes. We consider technical problems that impeded the function of the CRISPRa system at this haplotype block—for example, poor targeting or binding of the gRNAs at the selected PAM sequences.

For the common allele of rs34984145, a TFBS for BACH2 was predicted with a PWM matrix similarity of 83%. The antibody-specific EMSA gave evidence for BACH2 binding at the sequence of this SNP, but the luciferase reporter gene showed no differences of enhancer activity between alleles. This is why we considered that rs34984145 is not a causal variant of the association.

We conclude that rs4284742 and rs11084095 are functional variants, that the risk alleles reduce enhancer activity and binding affinity of the TFs MAFB and ERG, and that *SIGLEC5* is the target gene of these associations. We suggest a functional context of the associations with impaired regulation of endothelial homeostasis and healing of aseptic tissue injuries.

## Author Contributions

R. Mueller, A.S. Schaefer, contributed to conception, design, data acquisition, analysis, and interpretation, drafted and critically revised the manuscript; A. Chopra, contributed to conception, design, data analysis, and interpretation, critically revised the manuscript; H. Dommisch, contributed to data interpretation, critically revised the manuscript. All authors gave final approval and agree to be accountable for all aspects of the work.

## Supplemental Material

sj-docx-1-jdr-10.1177_00220345211049984 – Supplemental material for Periodontitis Risk Variants at SIGLEC5 Impair ERG and MAFB Binding Click here for additional data file. Supplemental material, sj-docx-1-jdr-10.1177_00220345211049984 for Periodontitis Risk Variants at SIGLEC5 Impair ERG and MAFB Binding by R. Mueller, A. Chopra, H. Dommisch and A.S. Schaefer in Journal of Dental Research

## References

[bibr1-00220345211049984] 1000 Genomes Project Consortium; Abecasis GR Altshuler D Auton A Brooks LD Durbin RM Gibbs RA Hurles ME McVean GA . 2010. A map of human genome variation from population-scale sequencing. Nature. 467(7319):1061–1073. 20981092 10.1038/nature09534 PMC3042601

[bibr2-00220345211049984] 1000 Genomes Project Consortium; Auton A Brooks LD Durbin RM Garrison EP Kang HM Korbel JO Marchini JL McCarthy S McVean GA , et al. 2015. A global reference for human genetic variation. Nature. 526(7571):68–74. 26432245 10.1038/nature15393 PMC4750478

[bibr3-00220345211049984] Ambrosini G Groux R Bucher P. 2018. PWMScan: a fast tool for scanning entire genomes with a position-specific weight matrix. Bioinformatics. 34(14):2483–2484. 29514181 10.1093/bioinformatics/bty127 PMC6041753

[bibr4-00220345211049984] Boyle EA Li YI Pritchard JK . 2017. An expanded view of complex traits: from polygenic to omnigenic. Cell. 169(7):1177–1186. 28622505 10.1016/j.cell.2017.05.038 PMC5536862

[bibr5-00220345211049984] Chen GY Tang J Zheng P Liu Y. 2009. CD24 and Siglec-10 selectively repress tissue damage-induced immune responses. Science. 323(5922):1722–1725. 19264983 10.1126/science.1168988 PMC2765686

[bibr6-00220345211049984] Crennell SJ Garman EF Laver WG Vimr ER Taylor GL . 1993. Crystal structure of a bacterial sialidase (from *Salmonella typhimurium* LT2) shows the same fold as an influenza virus neuraminidase. Proc Natl Acad Sci U S A. 90(21):9852–9856. 8234325 10.1073/pnas.90.21.9852 PMC47670

[bibr7-00220345211049984] Crocker PR Varki A. 2001. Siglecs, sialic acids and innate immunity. Trends Immunol. 22(6):337–342. 11377294 10.1016/s1471-4906(01)01930-5

[bibr8-00220345211049984] Drzeniek R . 1972. Viral and bacterial neuraminidases. Curr Top Microbiol Immunol. 59:35–74. 4144154 10.1007/978-3-642-65444-2_2

[bibr9-00220345211049984] ENCODE Project Consortium. 2012. An integrated encyclopedia of DNA elements in the human genome. Nature. 489(7414):57–74. 22955616 10.1038/nature11247 PMC3439153

[bibr10-00220345211049984] Gabriel SB Schaffner SF Nguyen H Moore JM Roy J Blumenstiel B Higgins J DeFelice M Lochner A Faggart M , et al. 2002. The structure of haplotype blocks in the human genome. Science. 296(5576):2225–2229. 12029063 10.1126/science.1069424

[bibr11-00220345211049984] Gemelli C Montanari M Tenedini E Zanocco Marani T Vignudelli T Siena M Zini R Salati S Tagliafico E Manfredini R , et al. 2006. Virally mediated MafB transduction induces the monocyte commitment of human CD34+ hematopoietic stem/progenitor cells. Cell Death Differ. 13(10):1686–1696. 16456583 10.1038/sj.cdd.4401860

[bibr12-00220345211049984] Gulino-Debrac D . 2013. Mechanotransduction at the basis of endothelial barrier function. Tissue Barriers. 1(2):e24180. 24665386 10.4161/tisb.24180 PMC3879236

[bibr13-00220345211049984] Heiss C Rodriguez-Mateos A Kelm M. 2015. Central role of eNOS in the maintenance of endothelial homeostasis. Antioxid Redox Signal. 22(14):1230–1242. 25330054 10.1089/ars.2014.6158 PMC4410282

[bibr14-00220345211049984] Jeong HW Hernandez-Rodriguez B Kim J Kim KP Enriquez-Gasca R Yoon J Adams S Scholer HR Vaquerizas JM Adams RH . 2017. Transcriptional regulation of endothelial cell behavior during sprouting angiogenesis. Nat Commun. 8(1):726. 28959057 10.1038/s41467-017-00738-7 PMC5620061

[bibr15-00220345211049984] Kalna V Yang Y Peghaire CR Frudd K Hannah R Shah AV Osuna Almagro L Boyle JJ Gottgens B Ferrer J , et al. 2019. The transcription factor ERG regulates super-enhancers associated with an endothelial-specific gene expression program. Cir Res. 124(9):1337–1349. 10.1161/CIRCRESAHA.118.313788 PMC6493686 30892142

[bibr16-00220345211049984] Kelly LM Englmeier U Lafon I Sieweke MH Graf T. 2000. MafB is an inducer of monocytic differentiation. EMBO J. 19(9):1987–1997. 10790365 10.1093/emboj/19.9.1987 PMC305687

[bibr17-00220345211049984] Kim K Kim JH Lee J Jin HM Kook H Kim KK Lee SY Kim N. 2007. MafB negatively regulates RANKL-mediated osteoclast differentiation. Blood. 109(8):3253–3259. 17158225 10.1182/blood-2006-09-048249

[bibr18-00220345211049984] Kim S Kim D Cho SW Kim J Kim JS . 2014. Highly efficient RNA-guided genome editing in human cells via delivery of purified Cas9 ribonucleoproteins. Genome Res. 24(6):1012–1019. 24696461 10.1101/gr.171322.113 PMC4032847

[bibr19-00220345211049984] Li W Zheng Q Meng H Chen D. 2020. Integration of genome-wide association study and expression quantitative trait loci data identifies AIM2 as a risk gene of periodontitis. J Clin Periodontol. 47(5):583–593. 32031269 10.1111/jcpe.13268

[bibr20-00220345211049984] Liu Y Chen GY Zheng P. 2009. CD24-Siglec G/10 discriminates danger- from pathogen-associated molecular patterns. Trends Immunol. 30(12):557–561. 19786366 10.1016/j.it.2009.09.006 PMC2788100

[bibr21-00220345211049984] Machiela MJ Chanock SJ . 2015. LDlink: a web-based application for exploring population-specific haplotype structure and linking correlated alleles of possible functional variants. Bioinformatics. 31(21):3555–3557. 26139635 10.1093/bioinformatics/btv402 PMC4626747

[bibr22-00220345211049984] Munz M Richter GM Loos BG Jepsen S Divaris K Offenbacher S Teumer A Holtfreter B Kocher T Bruckmann C , et al. 2019. Meta-analysis of genome-wide association studies of aggressive and chronic periodontitis identifies two novel risk loci. Eur J Hum Genet. 27(1):102–113. 30218097 10.1038/s41431-018-0265-5 PMC6303247

[bibr23-00220345211049984] Munz M Willenborg C Richter GM Jockel-Schneider Y Graetz C Staufenbiel I Wellmann J Berger K Krone B Hoffmann P , et al. 2017. A genome-wide association study identifies nucleotide variants at SIGLEC5 and DEFA1A3 as risk loci for periodontitis. Hum Mol Genet. 26(13):2577–2588. 28449029 10.1093/hmg/ddx151

[bibr24-00220345211049984] Pegon JN Kurdi M Casari C Odouard S Denis CV Christophe OD Lenting PJ . 2012. Factor VIII and von Willebrand factor are ligands for the carbohydrate-receptor Siglec-5. Haematologica. 97(12):1855–1863. 22733016 10.3324/haematol.2012.063297 PMC3685284

[bibr25-00220345211049984] Pepin M Mezouar S Pegon J Muczynski V Adam F Bianchini EP Bazaa A Proulle V Rupin A Paysant J , et al. 2016. Soluble Siglec-5 associates to PSGL-1 and displays anti-inflammatory activity. Sci Rep. 6:37953. 27892504 10.1038/srep37953 PMC5125011

[bibr26-00220345211049984] Pillai S Netravali IA Cariappa A Mattoo H. 2012. Siglecs and immune regulation. Annu Rev Immunol. 30:357–392. 22224769 10.1146/annurev-immunol-020711-075018 PMC3781015

[bibr27-00220345211049984] Rohs R Jin X West SM Joshi R Honig B Mann RS . 2010. Origins of specificity in protein-DNA recognition. Annu Rev Biochem. 79:233–269. 20334529 10.1146/annurev-biochem-060408-091030 PMC3285485

[bibr28-00220345211049984] Santos-Gallego CG. 2016. MafB and the role of macrophage apoptosis in atherosclerosis: a time to kill, a time to heal. Atherosclerosis. 252:194–196. 27338219 10.1016/j.atherosclerosis.2016.06.026

[bibr29-00220345211049984] Shungin D Haworth S Divaris K Agler CS Kamatani Y Keun Lee M Grinde K Hindy G Alaraudanjoki V Pesonen P , et al. 2019. Genome-wide analysis of dental caries and periodontitis combining clinical and self-reported data. Nat Commun. 10(1):2773. 10.1038/s41467-019-10630-1 PMC6591304 31235808

[bibr30-00220345211049984] Simeonov DR Gowen BG Boontanrart M Roth TL Gagnon JD Mumbach MR Satpathy AT Lee Y Bray NL Chan AY , et al. 2017. Discovery of stimulation-responsive immune enhancers with crispr activation. Nature. 549(7670):111–115. 28854172 10.1038/nature23875 PMC5675716

[bibr31-00220345211049984] Teumer A Holtfreter B Volker U Petersmann A Nauck M Biffar R Volzke H Kroemer HK Meisel P Homuth G , et al. 2013. Genome-wide association study of chronic periodontitis in a general German population. J Clin Periodontol. 40(11):977–985. 24024966 10.1111/jcpe.12154

[bibr32-00220345211049984] Timpson NJ Greenwood CMT Soranzo N Lawson DJ Richards JB . 2018. Genetic architecture: the shape of the genetic contribution to human traits and disease. Nat Rev Genet. 19(2):110–124. 29225335 10.1038/nrg.2017.101

[bibr33-00220345211049984] Tonnesen MG Feng X Clark RA . 2000. Angiogenesis in wound healing.J Investig Dermatol Symp Proc. 5(1):40–46. 10.1046/j.1087-0024.2000.00014.x 11147674

[bibr34-00220345211049984] Uhlen M Fagerberg L Hallstrom BM Lindskog C Oksvold P Mardinoglu A Sivertsson A Kampf C Sjostedt E Asplund A , et al. 2015. Proteomics. Tissue-based map of the human proteome. Science. 347(6220):1260419. 25613900 10.1126/science.1260419

[bibr35-00220345211049984] Weirauch MT Cote A Norel R Annala M Zhao Y Riley TR Saez-Rodriguez J Cokelaer T Vedenko A Talukder S , et al. 2013. Evaluation of methods for modeling transcription factor sequence specificity. Nat Biotechnol. 31(2):126–134. 23354101 10.1038/nbt.2486 PMC3687085

[bibr36-00220345211049984] Welter D MacArthur J Morales J Burdett T Hall P Junkins H Klemm A Flicek P Manolio T Hindorff L , et al. 2014. The NHGRI GWAS Catalog, a curated resource of SNP-trait associations. Nucleic Acids Res. 42(database issue):D1001–D1006. 10.1093/nar/gkt1229 PMC3965119 24316577

[bibr37-00220345211049984] Westra HJ Peters MJ Esko T Yaghootkar H Schurmann C Kettunen J Christiansen MW Fairfax BP Schramm K Powell JE , et al. 2013. Systematic identification of trans eQTLs as putative drivers of known disease associations. Nat Genet. 45(10):1238–1243. 24013639 10.1038/ng.2756 PMC3991562

[bibr38-00220345211049984] Wray NR Wijmenga C Sullivan PF Yang J Visscher PM . 2018. Common disease is more complex than implied by the core gene omnigenic model. Cell. 173(7):1573–1580. 29906445 10.1016/j.cell.2018.05.051

[bibr39-00220345211049984] Zeller T Wild P Szymczak S Rotival M Schillert A Castagne R Maouche S Germain M Lackner K Rossmann H , et al. 2010. Genetics and beyond—the transcriptome of human monocytes and disease susceptibility. PLoS One. 5(5):e10693. 20502693 10.1371/journal.pone.0010693 PMC2872668

