# Supplementary material for: Periodontitis Risk Variants at SIGLEC5 Impair ERG and MAFB Binding
Source: J Dent Res. 2021 Dec 2;101(5):551–8. doi: 10.1177/00220345211049984 (PMC9024020; doi:10.1177/00220345211049984)
Supplement: sj-docx-1-jdr-10.1177_00220345211049984 – Supplemental material for Periodontitis Risk Variants at SIGLEC5 Impair ERG and MAFB Binding [file sj-docx-1-jdr-10.1177_00220345211049984.docx]

**Appendix:**

**Periodontitis risk variants at *SIGLEC5* impair ERG and MAFB binding**

Ricarda Mueller^1^, Avneesh Chopra^1^, Henrik Dommisch^1^, Arne S. Schaefer^1^.

**Affiliations**:

^1^Department of Periodontology, Oral Medicine and Oral Surgery, Institute for Dental and Craniofacial Sciences, Charité – University Medicine Berlin, corporate member of Freie Universität Berlin, Humboldt-Universität zu Berlin, and Berlin Institute of Health, Berlin, 10117, Germany

**^2^**Freie Universität Berlin, Department of Biology, Chemistry and Pharmacy, Institute of Chemistry and Biochemistry

**Appendix Methods and Materials**

**Cell Culture and transfection**

*SIGLEC5* is strongly expressed in lymphocytes, including B cells. Raji cells were grown in RPMI-1640 Medium (ATCC 30-2001) supplemented with 10% fetal bovine serum and 1% Penicillin and Streptomycin to isolate the nuclear protein extract for the Electrophoretic Mobility Shift Assay (EMSA). To prepare the protein extract 2 x 10^7^ cells were grown and were washed twice with PBS. Cells were centrifuged at 200 x g for 10 minutes. The nuclear protein extracts were isolated using the Nuclear Extract Kit (Activemotif) according to the manufacturer’s instructions. Additionally, HeLa cells were used for luciferase-based reporter gene assays and CRISPR-dCas9 gene activation for their good transfection efficiencies.

HeLa cells were cultivated in cell growth medium (Earle’s MEM, 10% FCS, 2mM L-glutamine, 1% non-essential amino acids, 1% Penicillin and Streptomycin). One day before transfection, HeLa cells were seeded at 90,000 cells per well in 6-well tissue culture plates (TPP Techno Plastic Products). Cells were transfected using jetPEI transfection reagent (Polyplus transfection) according to the manufacturer’s instructions. For CRISPRa, HeLa cells were transfected in biological triplicates. Each Well of a 6-well-plate was transfected with 1 µg dCAS9-VP64_GFP [Plasmid #61422], 1 µg MS2-P65-HSF1_GFP [Plasmid #61423] and 1µg of a sgRNA(MS2), (all plasmids were obtained from Addgene, gifted by Feng Zhang). After 44 hours, HeLa cells were washed twice with PBS followed by cell disruption and RNA extraction using the RNeasy Mini Kit (Qiagen).

**Isolation of PBMCs**

Peripheral blood mononuclear cells (PBMC) including lymphocytes in which *SIGLEC5* is primarily expressed, were isolated from 10 mL whole blood, collected in sodium heparin treated tubes. Blood was diluted with an equal volume of 1 x PBS and mixed. The diluted blood was carefully layered over 15 mL Histopaque-1077 (Sigma Aldrich) and then centrifuged with very low acceleration and natural deceleration for 30 minutes at 400 x g. After centrifugation, the PBMC layer was transferred into a new tube and washed with 3 volumes of PBS followed by centrifugation at 350 x g for 10 minutes. The PBMC pellet was washed twice with PBS and subsequently the nuclear extract was isolated using the Nuclear Extract Kit (Activemotif) according to the manufacturer’s instructions.

**Selection of putative causal variant(s)**

We analyzed, if the associated SNPs were located at chromatin elements that correlated with regulatory functions of gene expression (Kreimer et al. 2017; Kwasnieski et al. 2014). These elements were open chromatin as determined by DNAse I hypersensitivity (DHS), epigenetic H3K27Ac and H3K4Me1 histone modifications, and transcription factor binding sites (TFBS) experimentally confirmed by ChIP-Seq, and chromatin state segmentation, that combines epigenomic data into a sequence of functional chromatin states (Mammana and Chung 2015).

**eQTL analysis**

The different alleles of a SNP may cause variation in expression levels of mRNAs, if they are located in a gene regulatory non-coding chromatin element (Rockman and Kruglyak 2006). These genomic locations are termed expression quantitative trait loci (eQTL). To annotate eQTL effects of the associated SNPs, we used the software tool QTLizer (Munz et al. 2020). The eQTLs of rs11084095 are listed in **Appendix Table 1** and the eQTLs of rs4284742 are listed in **Appendix Table 2**.

**Electrophoretic Mobility Shift Assay (EMSA)**

The double-stranded Biotin 3’ end-labeled and unlabeled oligonucleotides, which corresponded to each SNP allele and had a total length of 43 bp, were annealed with their complementary primers.

The oligonucleotide sequences were:

rs4284742-(G)-fw: CTGGTTCTTTCCACAGTCAC(C)AAGGACCACTCCATGCCCCTC;

rs4284742-(G)-rev:GAGGGGCATGGAGTGGTCCTT(G)GTGACTGTGGAAAGAACCAG;

rs4284742-(A)-fw: CTGGTTCTTTCCACAGTCAC(T)AAGGACCACTCCATGCCCCTC

rs4284742-(A)-rev: GAGGGGCATGGAGTGGTCCTT(A)GTGACTGTGGAAAGAACCAG

rs11084095-(A)-fw: CAATCTTTAGGTGATGCTAAA(A)GAAAGCTCGTGTGTGTTAGT

rs11084095-(A)-rev: ACTAACACACACGAGCTTTC(T)TTTAGCATCACCTAAAGATTG

rs11084095-(G)-fw: CAATCTTTAGGTGATGCTAAA(G)GAAAGCTCGTGTGTGTTAGT

rs11084095-(G)-rev: ACTAACACACACGAGCTTTC(C)TTTAGCATCACCTAAAGATTG

rs34984145-(A)-fw: TCCCAACTACTCGGAGGGCTG(A)GGCAGGAGAATGGCGTGAACC

rs34984145-(A)-rev: GGTTCACGCCATTCTCCTGCC(T)CAGCCCTCCGAGTAGTTGGGA

rs34984145-(T)-fw: TCCCAACTACTCGGAGGGCTG(T)GGCAGGAGAATGGCGTGAACC

rs34984145-(T)-rev: GGTTCACGCCATTCTCCTGCC(A)CAGCCCTCCGAGTAGTTGGGA

Used antibodies:

MAFB Polyclonal antibody (400 µg/mL); ERG monoclonal antibody (200 µg/mL); BACH2 polyclonal antibody (533 µg/mL)

**Luciferase reporter gene assay**

Genomic DNA (gDNA) was extracted from human cells using the AllPrep DNA/RNA/miRNA Universal Kit (Qiagen). The purified gDNA was used as a PCR-template. The DNA sequences spanning the putative causal SNPs were amplified by PCR to subsequently test their regulatory potential on reporter gene expression. Specifically, 75 bp up- and downstream of rs4284742, 65 bp up- and downstream of rs11084095 and 70 bp up- and downstream of rs34984145 were PCR amplified and cloned to the promoter of the firefly luciferase reporter gene of plasmid pGL4.24 (Promega).

The PCR product was amplified using Biozym Taq DNA Polymerase (Biozym) with forward and reverse primers containing the KpnI and XhoI restriction sites, respectively. The primer sequences were:

rs4284742-fw-primer 5’‑ ggccGGTACCCTCAAAGCAGTGAACAGACTTT-3’

rs4284742-rev-primer 5’- ggccGAGCTCATGCAGGAGTGGAAGGGTG-3’

rs11084095-fw-primer 5’‑ ggccGGTACCTGAGTTGTTTCCATTTGAGCCG

rs11084095-rev-primer 5’‑ ggccGAGCTCAAGATGCCCATTCACATGCC

rs34984145-fw-primer 5’‑ ggccGGTACCGTGAAACCCCGTCTCTACTAAA and

rs34984145-rev-primer 5’‑ cgaCTCGAGGCGATCTCCTCTCACTGCAA (enzyme restriction site underlined).

The PCR product was purified using QIAquick gel extraction kit (QIAGEN) and ligated to the XhoI and KpnI digested plasmid pGL4.24 (Promega). The modified plasmid was purified using the QIAprep Spin Miniprep Kit. The allele was exchanged with the Q5 site-directed mutagenesis kit (NEB) according to the manufacturer’s instructions. The primer sequences were:

rs4284742-Q5-fw-primer 5’‑ CCACAGTCACtAAGGACCACT-3’

rs4284742-Q5-rev-primer 5’- AAAGAACCAGACCACAGG-3’

rs11084095-fw-primer 5’‑ ACGAGCTTTCtTTTAGCATCAC

rs11084095-rev-primer 5’‑ GTGTGTTAGTACGGTGAG

rs34984145-fw-primer 5’‑ CGGAGGGCTGtGGCAGGAGAA

rs34984145-rev-primer 5’‑ AGTAGTTGGGACTACAGGCG

HeLa cells were co-transfected in triplicates using jetPEI transfection reagent according to the manufacturer’s instruction with either 2.7 µg luciferase reporter plasmid pGL4.24 carrying the rs4284742, rs11084095, or rs34984145 sequence cloned to the promoter of the luciferase reporter gene, and 0.3 µg *renilla* luciferase reporter vector (phRL-SV40, Promega). Additionally, as a control, HeLa cells were transfected with the empty pGL4.24 plasmid and 0.3 µg phRL-SV40 for 24 h. After 24 hours, the firefly and *renilla* luciferase activities were quantified using the Dual Luciferase Stop & Glo Reporter Assay System (Promega) with a luminometer (Orion II Microplate Luminometer, Berthold). The activities of the reporter gene assays were quantified as relative light units that were normalized as the ratio of firefly luciferase activity to *renilla* luciferase activity. Relative fold changes of normalized reporter gene activities were calculated using a T-Test.

**CRISPR-dCas9 activation**

All sgRNAs were synthesized (Metabion International AG) and annealed as described in (Ran et al. 2013). The ordered sgRNA sequences were:

gRNA-1-rs4284742-fw: CACCGTTATTTCCTGTCACATTAG;

gRNA-1-rs4284742-rev: AAACCTAATGTGACAGGAAATAAC;

gRNA-2-rs4284742-fw: CACCGCTCCTCCCCTGGCCTATGC;

gRNA-2-rs4284742-rev: AAACGCATAGGCCAGGGGAGGAGC;

gRNA-3-rs4284742-fw: CACCGACCACAGGAGGGATGGGAGA;

gRNA-3-rs4284742-rev: AAACTCTCCCATCCCTCCTGTGGTC;

gRNA-1-rs11084095-fw: CACCGCATCGCTGGGCATGTGAAT

gRNA-1-rs11084095-rev: AAACATTCACATGCCCAGCGATGC

gRNA-2-rs11084095-fw: CACCGTGTGCATCAATATGAGCTT

gRNA-2-rs11084095-rev: AAACAAGCTCATATTGATGCACAC

gRNA-3-rs11084095-fw: CACCGGCATAGATGAGAGTATAAA

gRNA-3-rs11084095-rev: AAACTTTATACTCTCATCTATGCC

scrambled-gRNA-fw: CACCGCACTACCAGAGCTAACTCA;

scrambled-gRNA-rev: AAACTGAGTTAGCTCTGGTAGTGC.

The double-stranded oligos were cloned into the *Bbs*I site of sgRNA(MS2) cloning backbone vector (Addgene Plasmid #61424). The modified plasmids were purified using the QIAprep Spin Miniprep Kit.

We designed three individual single-guide RNAs (sgRNA) that annealed 20-300 bp adjacent to each SNP using the online tool CRISPR-ERA (Liu et al. 2015). A scrambled sgRNA with no genomic target was used as a control.

**Quantitative Real-Time PCR**

To validate the effect of CRISPRa of the genomic location of rs4284742 and rs11084095 on *SIGLEC5* expression, 500 ng of total RNA of transfected HeLa cells were transcribed into cDNA using the High-Capacity cDNA Reverse Transcription Kit (Applied Biosystems). qRT-PCR was performed using SYBR Select Master Mix (Applied Biosystems) according to the manufacturer’s instructions. The expression of the genes *GAPDH* (fwd-primer 5’-GCA TCT TCT TTT GCG TCG; rev-primer 5’-TGT AAA CCA TGT AGT TGA GGT) and *SIGLEC5* (fwd-primer 5’-AAG GTC AAC TCC AGC TCA GC-3’, rev-primer 5’‑TTC GAT CTC CCT TGC AGC AG-3’) were quantified using the 2^‑ΔΔCT^ method as described in (Schmittgen and Livak 2008).


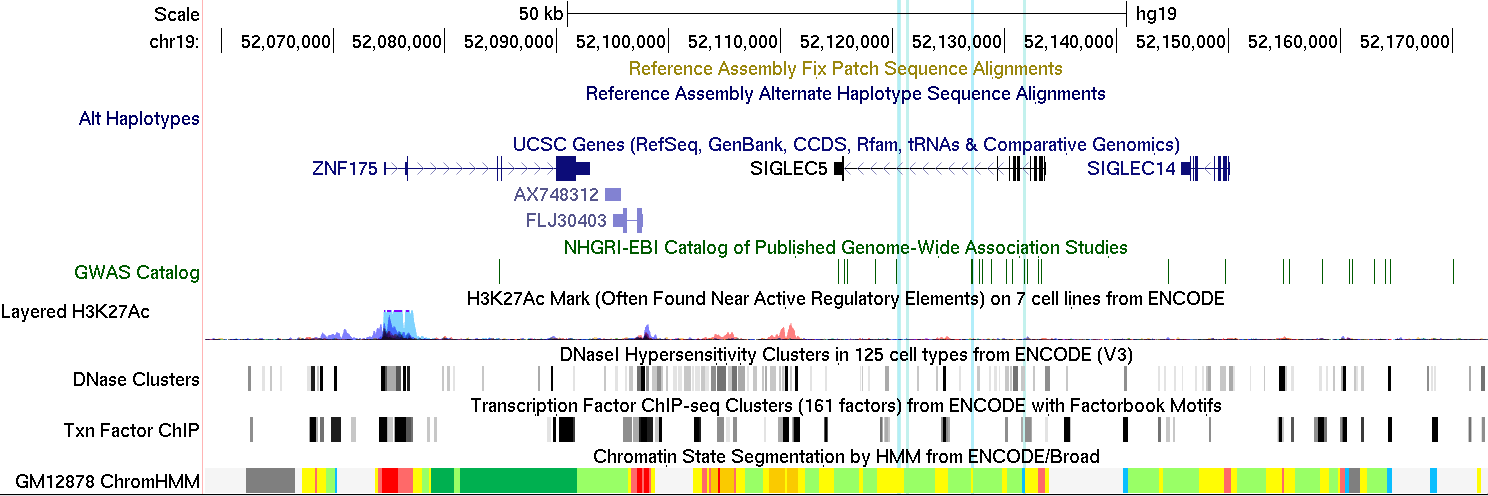


**Appendix Figure 1:** The genomic region at the *SIGLEC5* associations.

The SNPs that showed associations with increased periodontitis susceptibility at a genomewide significance level locate in introns at the gene *SIGLEC5*, which is flanked by the genes *SIGLEC14* and *ZNF175.* eQTL effects of rs4284742 and rs11084095 suggest *SIGLEC5* as the target gene of this association (**Appendix Table 1**). The different panels (DHS, TFBS, chromatin segmentation) are the same as described in Figure 1C.


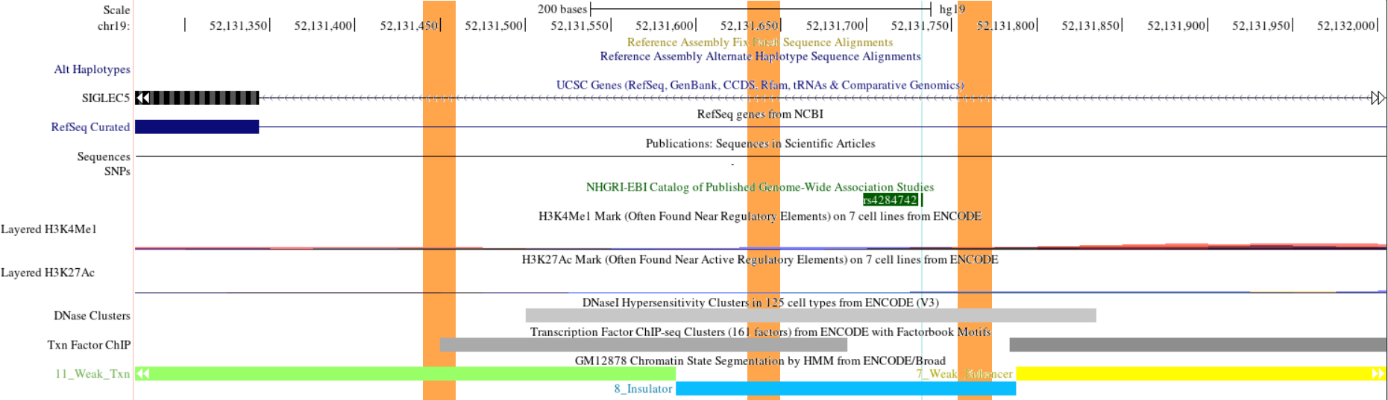


gRNA 1

gRNA 3

gRNA 2

**A**

**B**


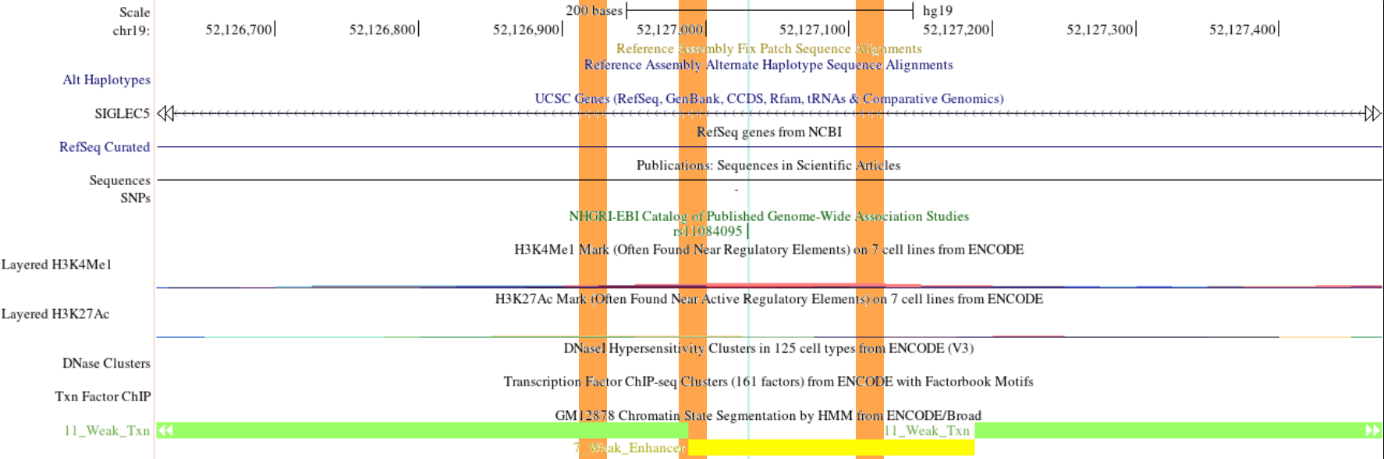


gRNA 3

gRNA 2

gRNA 1

**Appendix Figure 2: Position of gRNAs to test the genomic region at rs4284742 for its potential to activate *SIGLEC5* expression**

1. CRISPRa of the genomic region at rs4284742 induced *SIGLEC5* expression compared to the scrambled gRNA. gRNA 1 (chr19:52131631-52131649), gRNA 2 (chr19:52131441-52131459), and gRNA3 (chr19:52131754-52131773) induced *SIGLEC5* expression 380-fold upregulation (p = 0.00008), 137-fold (p = 0.03) and 80-fold (p = 0.0009), respectively (positions are given for genome build GRCh37/hg19)
2. CRISPRa of the genomic region at rs11084095 did not significantly induce *SIGLEC5* expression (gRNA 1 (chr19:52126982-52127000), gRNA 2 (chr19:52127105-52127123), and gRNA3 (52126912-52126930)).

**Appendix Table 1**. eQTL effects of rs428472 annotated by the software tool QTLizer.

| Index variant | LD-SNP (r^2^>0.8) | Affected Gene | Tissue | p-value | beta | Effect Allele | Non-Effect allele | Source |
| --- | --- | --- | --- | --- | --- | --- | --- | --- |
| rs4284742 | - | SIGLEC5 | Peripheral blood | 7.7e-14 | - | A | G | Blood eQTL Browser |
| rs4284742 | - | SHANK1 | Spleen | 6.9e-7 | -0.39 | G | A | GTEx v8 |
| rs4284742 | - | RPL9P33 | Whole blood | 0.0000022 | -0.18 | G | A | GTEx v8 |
| rs4284742 | - | SIGLEC14 | Pituitary | 0.00002 | -0.3 | G | A | GTEx v8 |
| rs4284742 | - | SIGLEC5 | Whole blood | 0.000033 | -0.11 | G | A | GTEx v8 |
| rs4284742 | - | NDUFA3 | Brain - Parietal lobe | 0.0043 | - | - | - | ScanDB |
| rs4284742 | - | ZNF813 | Brain - Cerebellum | 0.0045 | - | - | - | ScanDB |
| rs4284742 | - | ZNF787 | Brain - Parietal lobe | 0.0064 | - | - | - | ScanDB |
| rs4284742 | - | SYT5 | Brain - Parietal lobe | 0.0087 | - | - | - | ScanDB |
| rs4284742 | - | CD33 | Lymphoblastoid cell lines | 0.12 | - | - | - | seeQTL |

**Appendix Table 2**. eQTL effects of rs11084095 annotated by the software tool QTLizer.

| Index variant | LD-SNP (r^2^>0.8) | Affected Gene | Tissue | p-value | beta | Effect Allele | Non-Effect allele | Source |
| --- | --- | --- | --- | --- | --- | --- | --- | --- |
| rs11084095 | - | SIGLEC5 | Cells –  Monocytes | 6.4e-23 | - | - | - | Zeller et al. (PlosONE 2010) |
| rs11084095 | rs4801882 | SIGLEC5 | Cells –  Monocytes | 2.1e-16 | - | - | - | Zeller et al. (PlosONE 2010) |
| rs11084095 | - | SIGLEC5 | Adipose - Subcutaneous | 4.0e-16 | 0.4 | A | G | GTEx v8 |
| rs11084095 | rs12461706 | SIGLEC5 | Adipose - Subcutaneous | 4.0e-16 | 0.4 | T | A | GTEx v8 |
| rs11084095 | - | SIGLEC5 | Nerve - Tibial | 2.2e-14 | 0.4 | A | G | GTEx v8 |
| rs11084095 | rs12461706 | SIGLEC5 | Nerve - Tibial | 2.2e-14 | 0.4 | T | A | GTEx v8 |
| rs11084095 | - | SIGLEC5 | Artery - Tibial | 4.6e-14 | 0.38 | A | G | GTEx v8 |
| rs11084095 | rs12461706 | SIGLEC5 | Artery - Tibial | 6.2e-14 | 0.38 | T | A | GTEx v8 |
| rs11084095 | rs4801882 | SIGLEC5 | Nerve - Tibial | 1.7e-12 | 0.37 | A | G | GTEx v8 |
| rs11084095 | - | SIGLEC5 | Colon –  Sigmoid | 1.1e-11 | 0.48 | A | G | GTEx v8 |
| rs11084095 | rs12461706 | SIGLEC5 | Colon –  Sigmoid | 1.1e-11 | 0.48 | T | A | GTEx v8 |
| rs11084095 | rs4801882 | SIGLEC5 | Adipose - Subcutaneous | 4.0e-11 | 0.32 | A | G | GTEx v8 |
| rs11084095 | rs12461706 | SIGLEC5 | Cells - Macrophages | 1.2e-10 | -0.28 | A | T | The Cardiogenics Project |
| rs11084095 | - | SIGLEC5 | Cells - Macrophages | 1.4e-10 | -0.28 | G | A | The Cardiogenics Project |
| rs11084095 | - | SIGLEC5 | Esophagus - Muscularis | 3.0e-10 | 0.37 | A | G | GTEx v8 |
| rs11084095 | rs12461706 | SIGLEC5 | Esophagus - Muscularis | 3.0e-10 | 0.37 | T | A | GTEx v8 |
| rs11084095 | - | SIGLEC5 | Adipose –  Visceral (Omentum) | 1.7e-9 | 0.23 | A | G | GTEx v8 |
| rs11084095 | rs4801882 | SIGLEC5 | Artery - Tibial | 2.6e-9 | 0.29 | A | G | GTEx v8 |
| rs11084095 | rs12461706 | SIGLEC5 | Adipose –  Visceral (Omentum) | 3.1e-9 | 0.23 | T | A | GTEx v8 |
| rs11084095 | - | SIGLEC5 | Artery - Aorta | 1.2e-8 | 0.29 | A | G | GTEx v8 |
| rs11084095 | rs12461706 | SIGLEC5 | Artery - Aorta | 1.8e-8 | 0.29 | T | A | GTEx v8 |
| rs11084095 | - | SIGLEC5 | Colon - Transverse | 3.5e-8 | 0.31 | A | G | GTEx v8 |
| rs11084095 | rs12461706 | SIGLEC5 | Colon - Transverse | 3.5e-8 | 0.31 | T | A | GTEx v8 |
| rs11084095 | - | SIGLEC5 | Stomach | 3.8e-8 | 0.33 | A | G | GTEx v8 |
| rs11084095 | rs12461706 | SIGLEC5 | Stomach | 3.8e-8 | 0.33 | T | A | GTEx v8 |
| rs11084095 | rs4801882 | LLNLR-470E3.1 | Whole blood | 5.2e-8 | -0.24 | A | G | GTEx v8 |
| rs11084095 | - | LLNLR-470E3.1 | Whole blood | 8.0e-8 | -0.25 | A | G | GTEx v8 |
| rs11084095 | rs12461706 | LLNLR-470E3.1 | Whole blood | 8.0e-8 | -0.25 | T | A | GTEx v8 |
| rs11084095 | rs4801882 | SIGLEC5 | Colon –  Sigmoid | 1.0e-7 | 0.37 | A | G | GTEx v8 |
| rs11084095 | rs4801882 | SIGLEC5 | Esophagus - Muscularis | 1.1e-7 | 0.31 | A | G | GTEx v8 |
| rs11084095 | rs12461706 | SIGLEC5 | Breast - Mammary  tissue | 1.2e-7 | 0.34 | T | A | GTEx v8 |
| rs11084095 | - | SIGLEC5 | Breast – Mammary  tissue | 1.3e-7 | 0.34 | A | G | GTEx v8 |
| rs11084095 | rs4801882 | SIGLEC5 | Breast - Mammary  tissue | 1.3e-7 | 0.33 | A | G | GTEx v8 |
| rs11084095 | rs4801882 | SIGLEC5 | Artery - Aorta | 1.8e-7 | 0.25 | A | G | GTEx v8 |
| rs11084095 | rs12461706 | SIGLEC5 | Skin - Sun exposed  (Lower leg) | 2.4e-7 | 0.25 | T | A | GTEx v8 |
| rs11084095 | - | SIGLEC5 | Skin - Sun exposed  (Lower leg) | 3.0e-7 | 0.25 | A | G | GTEx v8 |
| rs11084095 | - | SIGLEC5 | Adrenal gland | 3.3e-7 | 0.45 | A | G | GTEx v8 |
| rs11084095 | rs12461706 | SIGLEC5 | Adrenal gland | 3.3e-7 | 0.45 | T | A | GTEx v8 |
| rs11084095 | rs4801882 | SIGLEC5 | Cells - Macrophages | 4.3e-7 | -0.21 | G | A | The Cardiogenics Project |
| rs11084095 | rs4801882 | SIGLEC5 | Skin - Sun exposed (Lower leg) | 5.7e-7 | 0.24 | A | G | GTEx v8 |
| rs11084095 | rs4801882 | SIGLEC5 | Adipose –  Visceral (Omentum) | 8.3e-7 | 0.18 | A | G | GTEx v8 |
| rs11084095 | rs4801882 | SIGLEC14 | Whole blood | 9.2e-7 | -0.21 | A | G | GTEx v8 |
| rs11084095 | - | SIGLEC5 | Heart - Atrial appendage | 9.7e-7 | 0.26 | A | G | GTEx v8 |
| rs11084095 | rs12461706 | SIGLEC5 | Heart - Atrial appendage | 9.7e-7 | 0.26 | T | A | GTEx v8 |
| rs11084095 | rs4801882 | B4GALNT1 | Cells - Macrophages | 1.5e-6 | 0.06 | G | A | The Cardiogenics Project |
| rs11084095 | - | SHBG | Cells - Macrophages | 2.2e-6 | 0.05 | G | A | The Cardiogenics Project |
| rs11084095 | rs12461706 | SHBG | Cells - Macrophages | 2.7e-6 | 0.05 | A | T | The Cardiogenics Project |
| rs11084095 | - | SIGLEC5 | Lung | 3.0e-6 | 0.19 | A | G | GTEx v8 |
| rs11084095 | rs12461706 | SIGLEC5 | Lung | 3.0e-6 | 0.19 | T | A | GTEx v8 |
| rs11084095 | - | SIGLEC14 | Whole blood | 3.5e-6 | -0.21 | A | G | GTEx v8 |
| rs11084095 | rs12461706 | SIGLEC14 | Whole blood | 3.5e-6 | -0.21 | T | A | GTEx v8 |
| rs11084095 | - | ZNF480 | Ovary | 5.8e-6 | 0.41 | A | G | GTEx v8 |
| rs11084095 | rs12461706 | ZNF480 | Ovary | 5.8e-6 | 0.41 | T | A | GTEx v8 |
| rs11084095 | rs4801882 | PSMB6 | Cells - Macrophages | 7.7e-6 | 0.04 | G | A | The Cardiogenics Project |
| rs11084095 | - | SIGLEC5 | Esophagus – Gastroeso-phageal  junction | 7.7e-6 | 0.3 | A | G | GTEx v8 |
| rs11084095 | rs12461706 | SIGLEC5 | Esophagus – Gastroeso-phageal  junction | 7.7e-6 | 0.3 | T | A | GTEx v8 |
| rs11084095 | rs12461706 | EMC6 | Cells - Macrophages | 7.9e-6 | 0.05 | A | T | The Cardiogenics Project |
| rs11084095 | rs4801882 | SIGLEC5 | Pituitary | 9.1e-6 | 0.32 | A | G | GTEx v8 |
| rs11084095 | - | EMC6 | Cells - Macrophages | 9.2e-6 | 0.05 | G | A | The Cardiogenics Project |
| rs11084095 | - | SIGLEC5 | Muscle skeletal | 1.0e-5 | 0.22 | A | G | GTEx v8 |
| rs11084095 | rs4801882 | SIGLEC5 | Adrenal gland | 1.2e-5 | 0.37 | A | G | GTEx v8 |
| rs11084095 | rs12461706 | SIGLEC5 | Muscle skeletal | 1.4e-5 | 0.22 | T | A | GTEx v8 |
| rs11084095 | rs12461706 | SIGLEC5 | Esophagus - Mucosa | 1.6e-5 | 0.22 | T | A | GTEx v8 |
| rs11084095 | rs4801882 | SIGLEC5 | Heart - Atrial appendage | 1.6e-5 | 0.23 | A | G | GTEx v8 |
| rs11084095 | - | SIGLEC5 | Esophagus - Mucosa | 1.9e-5 | 0.22 | A | G | GTEx v8 |
| rs11084095 | - | SIGLEC5 | Prostate | 1.9e-5 | 0.34 | A | G | GTEx v8 |
| rs11084095 | - | SIGLEC5 | Skin – Not  sun exposed (Suprapubic) | 1.9e-5 | 0.23 | A | G | GTEx v8 |
| rs11084095 | rs12461706 | SIGLEC5 | Prostate | 1.9e-5 | 0.34 | T | A | GTEx v8 |
| rs11084095 | rs12461706 | SIGLEC5 | Skin - Not sun exposed (Suprapubic) | 1.9e-5 | 0.23 | T | A | GTEx v8 |
| rs11084095 | rs4801882 | SIGLEC5 | Colon - Transverse | 2.3e-5 | 0.23 | A | G | GTEx v8 |
| rs11084095 | rs4801882 | SIGLEC5 | Skin - Not  sun exposed (Suprapubic) | 4.9e-5 | 0.21 | A | G | GTEx v8 |
| rs11084095 | - | NDUFA3 | Brain –  Parietal lobe | 2.6e-4 | - | - | - | ScanDB |
| rs11084095 | - | SIGLEC5 | Peripheral  blood | 3.3e-4 | - | A | G | Blood eQTL Browser |
| rs11084095 | rs4801882 | NDUFA3 | Brain –  Parietal lobe | 4.5e-4 | - | - | - | ScanDB |
| rs11084095 | - | ZNF416 | Brain –  Parietal lobe | 2.1e-3 | - | - | - | ScanDB |
| rs11084095 | rs4801882 | ZNF416 | Brain –  Parietal lobe | 2.8e-3 | - | - | - | ScanDB |
| rs11084095 | rs4801882 | ZNF321P | Brain - Cerebellum | 3.1e-3 | - | - | - | ScanDB |
| rs11084095 | - | ZNF784 | Brain –  Parietal lobe | 3.9e-3 | - | - | - | ScanDB |
| rs11084095 | - | ZNF321P | Brain - Cerebellum | 4.3e-3 | - | - | - | ScanDB |
| rs11084095 | rs4801882 | SIGLEC5 | Cells –  Lympho-  blastoid  cell lines | 7.0e-2 | - | - | - | seeQTL |

**References**

Kreimer A, Zeng H, Edwards MD, Guo Y, Tian K, Shin S, Welch R, Wainberg M, Mohan R, Sinnott-Armstrong NA et al. 2017. Predicting gene expression in massively parallel reporter assays: A comparative study. Hum Mutat.

Kwasnieski JC, Fiore C, Chaudhari HG, Cohen BA. 2014. High-throughput functional testing of encode segmentation predictions. Genome Res. 24(10):1595-1602.

Liu H, Wei Z, Dominguez A, Li Y, Wang X, Qi LS. 2015. Crispr-era: A comprehensive design tool for crispr-mediated gene editing, repression and activation. Bioinformatics. 31(22):3676-3678.

Mammana A, Chung HR. 2015. Chromatin segmentation based on a probabilistic model for read counts explains a large portion of the epigenome. Genome Biol. 16:151.

Munz M, Wohlers I, Simon E, Reinberger T, Busch H, Schaefer AS, Erdmann J. 2020. Qtlizer: Comprehensive qtl annotation of gwas results. Sci Rep. 10(1):20417.

Ran FA, Hsu PD, Wright J, Agarwala V, Scott DA, Zhang F. 2013. Genome engineering using the crispr-cas9 system. Nat Protoc. 8(11):2281-2308.

Rockman MV, Kruglyak L. 2006. Genetics of global gene expression. Nat Rev Genet. 7(11):862-872.

Schmittgen TD, Livak KJ. 2008. Analyzing real-time pcr data by the comparative c(t) method. Nat Protoc. 3(6):1101-1108.
